# A wearable monitoring system for running gait analysis by diffusion transformer

**DOI:** 10.1371/journal.pone.0341066

**Published:** 2026-01-23

**Authors:** Xiaoxue Hu, Guoyu Wang, Guodong Ma

**Affiliations:** 1 Department of Leisure Service and Sports, Paichai University, Daejeon, Korea; 2 Department of Physical Education, Pukyong National University, Busan, Republic of South Korea; 3 Human Movement Science College, Jilin Sport University, Changchun, Jilin, China; Sunway University, MALAYSIA

## Abstract

Conventional wearable monitoring devices often suffer from insufficient data accuracy and low posture recognition rates, making them inadequate for the demands of professional sports health monitoring. To address these issues, this study proposes a wearable monitoring system for running gait analysis based on the Diffusion Transformer (DiT). The system aims to achieve high-precision running posture recognition and real-time motion monitoring through multi-sensor data fusion and advanced deep learning architecture. First, a wearable system was developed using a nine-axis Micro-Electro-Mechanical System (MEMS) inertial sensor and an UltraWide Band (UWB) positioning module. Data quality was enhanced through sensor calibration, noise compensation, and an adaptive filtering algorithm. Then, a DiT-LSTM running posture recognition model was constructed by integrating the DiT with a Long Short-Term Memory (LSTM) network to perform posture recognition within the wearable system. Experimental results show that on the Human3.6M dataset, the DiT-LSTM model achieved an accuracy of 97.54%, a precision of 97.61%, a recall of 97.73%, an F1-score of 97.58%, and an Area Under the Curve (AUC) of 98.61%. On the HumanEva dataset, the model attained an accuracy of 96.39%, a precision of 96.47%, a recall of 96.8%, an F1-score of 96.92%, and an AUC of 97.9%, all outperforming other algorithms. The complexity assessment showed that DiT-LSTM reached 14.7 GFLOPs on the Human3.6M dataset, with a per-epoch training time of 62.3 seconds, while its per-sample inference latency was only 9.4 ms, meeting real-time monitoring requirements. In the UWB-based position drift correction experiment, Sample 1 achieved mean errors of 0.637 m, 0.581 m, and 0.349 m on the X/Y/Z axes, with corresponding RMSE values of 0.041 m, 0.023 m, and 0.025 m, demonstrating high positioning accuracy and stability. By combining multimodal sensors with the DiT-LSTM model, the study offers reliable technical support for running gait analysis, injury prevention, and personalized training guidance.

## Introduction

In recent years, the rapid advancement of wearable technology has highlighted the potential of multimodal sensor-based motion monitoring systems in areas such as sports science, rehabilitation medicine, and smart athletics [1]. As a fundamental form of human locomotion, running demands accurate analysis of gait characteristics, including joint angles, stride symmetry, and center-of-mass (CoM) trajectories, which are essential for injury prevention, performance optimization, and correction of pathological gait patterns [2,3]. Although traditional motion capture systems offer millimeter-level precision, their high cost, dependency on controlled environments, and lack of portability limit their practical application [4]. In contrast, inertial sensor-based solutions are more compact and wearable but are often hindered by issues such as sensor drift, noise interference, and cumulative pose estimation errors during complex movements [5]. Ultra-Wideband (UWB) positioning modules can mitigate some positional drift; however, their performance is still compromised in challenging environments involving dynamic occlusion or sudden magnetic field disturbances [6]. While deep learning has driven notable progress in action recognition, conventional models like Long Short-Term Memory (LSTM) networks and convolutional neural networks (CNNs) are sensitive to temporal noise and struggle to model global spatiotemporal relationships across joints [7]. Transformer-based architectures improve long-range dependency modeling through self-attention mechanisms, but their high computational demands and limited ability to capture localized motion nuances pose challenges for deployment in embedded systems [8]. As such, there is an urgent need for a wearable gait analysis system that combines high accuracy, low power consumption, and robust noise resilience.

This study addresses that need by integrating Micro-Electro-Mechanical System (MEMS) inertial sensors with UWB positioning modules to enhance data quality. It further introduces the DiT-LSTM model, a hybrid framework that combines the powerful feature extraction capabilities of the Diffusion Transformer (DiT) with the temporal modeling efficiency of LSTM, to enable high-precision running gait recognition and real-time motion monitoring. The core innovation lies in this novel integration of DiT and LSTM, which significantly improves recognition accuracy and stability. Additionally, the MEMS-UWB fusion system enhances data reliability and real-time responsiveness, enabling the proposed solution to effectively support complex motion monitoring in real-world scenarios.

## Literature review

In recent years, deep learning has emerged as a key breakthrough in the field of motion posture recognition [9]. Malik et al. (2023) proposed an efficient multi-view interaction-level action recognition system based on a CNN-LSTM architecture. By extracting 2D skeletal data instead of using raw image inputs, their method significantly reduced computational complexity. Experiments demonstrated recognition accuracies of 94.4% on the Motion Capture Activity Dataset (MCAD) and 91.67% on the INRIA Xmas Multi-view Action Dataset (IXMAS), outperforming traditional approaches [10]. Li and Ullah (2023) introduced a deep learning method that integrated CNNs with graph convolutional networks to recognize the actions of football players from videos and images. Their model effectively captured spatiotemporal posture features and achieved an accuracy of 97.4% in recognizing 17 complex football movements, demonstrating a significant performance advantage over previous studies [11]. Munsif et al. (2024) proposed that an action and interaction recognition network based on a convolutional block attention mechanism could effectively identify human actions and interactions under both infrared and visible light conditions [12]. Pabba et al. (2024) demonstrated that OpenPose, an intelligent system leveraging multiple visual cues, could automatically monitor and provide feedback on both individual and group behaviors in classroom settings by integrating students’ body posture, mobile phone usage, and facial expressions [13]. Manakitsa et al. (2024) emphasized that recurrent neural networks (RNNs) and three-dimensional (3D) CNNs within deep learning frameworks could efficiently extract features from video or sensor data, enabling accurate recognition and classification of human activities. These methods were widely applied in healthcare, security surveillance, and sports analytics [14]. Lee et al. (2024) proposed a posture pattern mining method that combined EfficientNet with a Transformer-based mechanism to perform time-series-based human action classification. Their findings indicated that this approach improved recognition accuracy while maintaining real-time performance [15].

In summary, although significant progress has been made in motion posture recognition, existing studies still face several limitations. Many approaches rely on complex computational resources, resulting in high computational overhead and reduced efficiency in real-time applications. Furthermore, while some methods perform well on specific datasets, their generalization to other scenarios is often limited, highlighting the need for improved adaptability and robustness. To address these challenges, this study proposes a running gait recognition model that combines DiT and LSTM architectures. By integrating data from multiple sensors and leveraging advanced deep learning structures, the model effectively reduces computational complexity while achieving high-precision gait recognition across multiple datasets. Additionally, the incorporation of a UWB correction algorithm further enhances positioning accuracy, system stability, and real-time performance.

## Theoretical foundation and DiT-based running gait detection system

### Human posture kinematics

**Fig 1:**
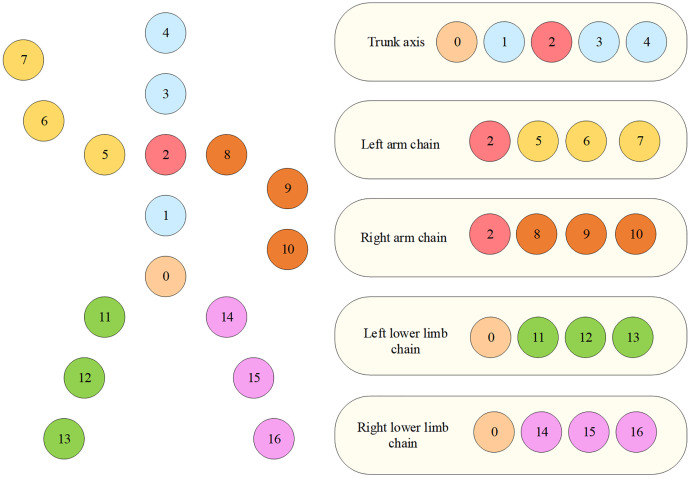
Human Skeletal Model.

Human posture kinematics is the study of the spatial positioning, joint angle variations, and motion trajectories of body parts in both static and dynamic states. At its core, this discipline focuses on the quantitative analysis of the coordinated interactions among the skeletal, muscular, and joint systems [16]. During movement, the human body achieves precise coordination of multi-degree-of-freedom joints—such as the hip, knee, and ankle—through neuromuscular control, forming distinct movement patterns, such as the stance and swing phases within the gait cycle. Kinematic parameters, including joint angles, angular velocity, limb displacement, and CoM trajectory, directly reflect movement efficiency, energy expenditure, and potential injury risks [17]. This study classifies 17 key joint points into five functional chains based on anatomical structure and the coordinated roles of the human musculoskeletal system: left arm chain, trunk axis, right arm chain, left lower limb chain, and right lower limb chain. The human skeletal model is illustrated in Fig 1 [18].

Let $q_{parent}$ and $q_{child}$ denote the quaternion representations of the parent and child joints, respectively, with w indicating the dimensionality. The joint angle is computed as shown in Equation (1) [19]:


$$\theta_{joint}=𝐚𝐫𝐜𝐜𝐨𝐬\left(2(q_{parent}⊗q_{child}^{-1}{)}_{w}^{2}-1\right)$$ (1)

$\theta_{joint}$ represents the flexion/extension angle of the joint, reflecting the spatial relative motion between adjacent limb segments. The operator ⊗ denotes quaternion multiplication, and $q_{child}^{-1}$ is the inverse of the child joint quaternion.

By calculating joint angles, local limb motion details can be captured. Building upon this, the CoM trajectory of the human body can be estimated to describe the global movement pattern. The CoM trajectory is calculated as shown in Equation (2) [20]:


$$\begin{array}{c} {𝐩}_{CoM}(t)=\frac{1}{N}\sum {\mathrm{\backslash nolimits}}_{i=1}^{N}\left[\begin{array}{c} {𝐩}_{i}(t)+{𝐑}_{i}\left(t\right){𝐫}_{i}^{CoM} \end{array}\right] \end{array}$$ (2)

${𝐩}_{i}\left(t\right)$ denotes the 3D positional coordinates of the iii-th joint at time t; ${𝐑}_{i}\left(t\right)$ is the rotation matrix corresponding to the sensor; ${𝐫}_{i}^{CoM}$ is the fixed offset vector from the sensor mounting point to the CoM; and N is the total number of joints.

Accurate estimation of the CoM trajectory requires not only the fusion of multi-source sensor data but also the segmentation of gait cycles through temporal alignment methods to analyze the spatiotemporal characteristics of different movement phases. Gait cycle phase segmentation is computed as shown in Equation (3) [21]:


$$D(i,j)=min\left\{ \begin{array}{c} D(i-1,j)+d({𝐯}_{i},{𝐯}_{j})\\ D(i,j-1)+d({𝐯}_{i},{𝐯}_{j})\\ D(i-1,j-1)+2d({𝐯}_{i},{𝐯}_{j}) \end{array}\right.\;$$ (3)

D(i,j) is the accumulated distance between the *i*-th frame of the test sequence and the *j*-th frame of the template sequence; $d({𝐯}_{i},{𝐯}_{j})$ represents the similarity between the output feature vectors ${𝐯}_{i}$ and ${𝐯}_{j}$; and ${𝐯}_{i}$ denotes the spatiotemporal feature vector at frame i.

### Wearable detection system based on inertial sensors and UWB positioning

This study utilizes a hybrid system comprising a 9-axis MEMS inertial sensor and a UWB positioning module. By applying an Extended Kalman Filter (EKF) in conjunction with the Rauch–Tung–Striebel (RTS) smoothing algorithm, multi-source data fusion is achieved to mitigate sensor drift and non-line-of-sight (NLOS) interference from the UWB module.

The 9-axis MEMS inertial sensor integrates a tri-axial accelerometer, gyroscope, and magnetometer, enabling the simultaneous acquisition of linear acceleration, angular velocity, and magnetic field data. These measurements provide comprehensive support for attitude estimation. The accelerometer senses linear acceleration along the X, Y, and Z axes via micro-mechanical structures; the gyroscope measures angular velocity around three axes using the Coriolis effect; and the magnetometer detects Earth’s magnetic field vector through either the Hall effect or magneto-resistive technology. After fusing these sensor readings, the device’s absolute orientation in 3D space can be reconstructed using quaternion or rotation matrix representations [22].

During sensor error modeling and calibration, let $S_{x}$, $S_{y}$, and $S_{z}$ denote the scaling factors along the three axes; $X_{1}$, $Y_{1}$, and $Z_{1}$ are the ideal measurement values; and $X_{2}$, $Y_{2}$, and $Z_{2}$ are the actual outputs. The scale factor error model is expressed as shown in Equation (4) [23]:


$$\left[\begin{array}{c} X_{2}\\ Y_{2}\\ Z_{2} \end{array}\right]=[\begin{array}{ccc} S_{x} & 0 & 0\\ 0 & S_{y} & 0\\ 0 & 0 & S_{z} \end{array}]\left[\begin{array}{c} X_{1}\\ Y_{1}\\ Z_{1} \end{array}\right]$$ (4)

Zero bias error, caused by hardware circuit offset, is modeled as shown in Equation (5):


$$\left[\begin{array}{c} X\\ Y\\ Z \end{array}\right]=\left[\begin{array}{c} X_{2}\\ Y_{2}\\ Z_{2} \end{array}\right]+\left[\begin{array}{c} b_{x}\\ b_{y}\\ b_{z} \end{array}\right]$$ (5)

$b_{x}$, $b_{y}$, and $b_{z}$ represent the zero-bias errors along the three axes.

Sensor calibration is fundamental to accurate attitude estimation. Due to the high-frequency dynamic characteristics of the gyroscope, compensation using the Earth’s rotational model is necessary. The angular velocity output of the gyroscope is calculated as shown in Equation (6) [24]:


$$\omega_{bi}^{b}=R_{b}^{n}\left(-\frac{V_{N}}{R+h}\omega_{ie}𝐜𝐨𝐬\emptyset +\frac{V_{E}}{R+h}\omega_{ie}𝐬𝐢𝐧\emptyset \right)+\omega_{nn}^{b}$$ (6)

$\omega_{bi}^{b}$ represents the angular velocity of the body frame relative to the inertial frame; R denotes the Earth’s radius; $\omega_{ie}$ is the Earth’s rotational angular velocity; $V_{N}$ and $V_{E}$ are the northward and eastward velocities of the body, respectively; and h is the body’s altitude. $\omega_{nn}^{b}$ is the angular velocity of the navigation frame relative to the body frame, expressed in body coordinates; ∅ is the latitude at the body’s current position.

This study employs a UWB positioning module to enhance the performance of a 9-axis MEMS inertial sensor. The developed MEMS-UWB system uses symmetric double-sided two-way ranging (SDS-TWR) to eliminate clock offset, while the RTS smoothing algorithm is applied to suppress NLOS outlier errors. The UWB positioning module uses nanosecond-scale narrow pulses (bandwidth ≥ 500 MHz) to achieve centimeter-level ranging accuracy. Its core principles include Time of Arrival (ToA), Time Difference of Arrival (TDoA), and SDS-TWR [25].

In UWB ranging and clock error suppression, let $b_{i}$ denote the coordinates of the i-th UWB anchor; p is the position of the tag; Δτ means the clock offset between the anchor and the tag; $\delta_{i}$ refers to the error introduced by NLOS and multipath effects; and $\nu_{i}$ is the Gaussian measurement noise. The SDS-TWR ranging model is computed as shown in Equation (7):


$$TOF_{i}=\frac{∥b_{i}-p∥}{c}+\Delta \tau +\delta_{i}+\nu_{i}$$ (7)

Based on the MEMS-UWB multi-sensor fusion positioning system, the human kinematic equations and state models are expressed in Equations (8) and (9) [26]:


$$v_{k}=v_{k-1}+\Delta t\times a_{k}$$ (8)

$$p_{k}=p_{k-1}+v_{k-1}\Delta t+0.5\Delta t^{2}a_{k}$$ (9)

$v_{k}$ and $p_{k}$ represent the velocity and position vectors, respectively; $a_{k}$ denotes the gravity-compensated linear acceleration of the carrier; $v_{k-1}$ and $p_{k-1}$ are the velocity and position vectors at time step k−1; and Δt is the time interval.

To enhance the real-time performance of the MEMS-UWB system, the EKF is introduced. The EKF equations are shown in Equations (10) and (11) [27]:


$$x_{k}=F_{k}x_{k-1}+B_{k}u_{k}+w_{k}$$ (10)

$$z_{k}=C_{k}x_{k}+v_{k}$$ (11)

$x_{k}$ is the state vector; $F_{k}$ is the state transition matrix; $B_{k}$ is the input matrix; $w_{k}$ is the process noise; $u_{k}$ is the control input; $z_{k}$ is the observation vector; $C_{k}$ is the observation matrix; and $v_{k}$ is the UWB position noise.

The RTS smoothing algorithm is applied for backward correction of the MEMS-UWB system. The backward smoothed state estimation is calculated using Equations (12) and (13) [28]:


$$K_{k}^{s}=P_{k}^{+}F_{k}(P_{k+1}^{-}{)}^{-1}$$ (12)

$$x_{k}^{s}=x_{k}^{+}+K_{k}^{s}(x_{k+1}^{s}-x_{k+1}^{-})$$ (13)

$K_{k}^{s}$: the RTS smoothing gain at time k; $P_{k}^{+}$: the a posteriori covariance matrix at time k; $P_{k+1}^{-}$: the a priori covariance matrix at time k+1; $x_{k}^{s}$: the smoothed state estimate at time k; $x_{k}^{+}$: the a posteriori state estimate at time kkk; $x_{k+1}^{-}$: the a priori state estimate at time k+1; and $x_{k+1}^{s}$: the smoothed state estimate at time k+1.

### Running gait recognition model based on DiT

The Transformer is a deep learning architecture built upon the self-attention mechanism. By leveraging this mechanism, it efficiently captures long-range dependencies within input sequences. The core calculation is shown in Equation (14):


$$𝐀𝐭𝐭𝐞𝐧𝐭𝐢𝐨𝐧(Q,K,V)=𝐬𝐨𝐟𝐭𝐦𝐚𝐱\left(\frac{QK^{T}}{\sqrt{d_{k}}}\right)V$$ (14)

Q,K,V represent the query, key, and value matrices, respectively; and $d_{k}$ is the dimensionality of the key vectors. The self-attention mechanism computes the similarity between queries and keys, then uses this to generate weighted combinations of the value vectors, thereby achieving dynamic information integration.

**Fig 2:**
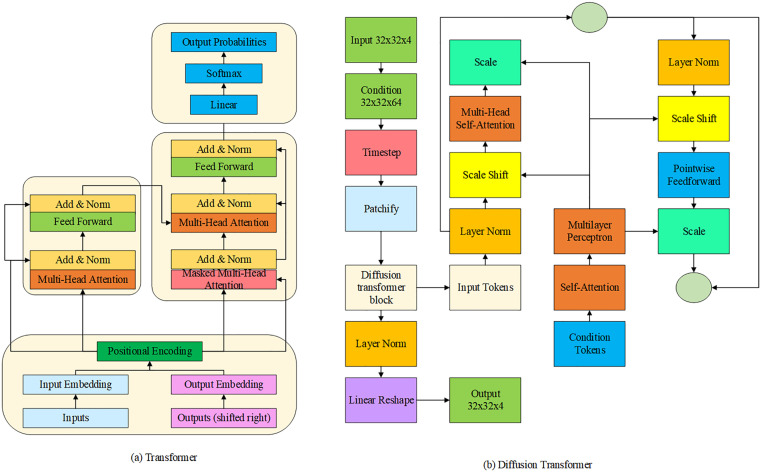
Architectures of Transformer and DiT.

The DiT is a model that integrates the Transformer architecture with diffusion models. It utilizes the powerful sequence modeling capabilities of Transformers to learn complex data distributions. The DiT has demonstrated outstanding performance in tasks such as image and audio generation, where it can generate high-quality samples while effectively leveraging contextual information and long-range dependencies. The architectures of both the standard Transformer and the DiT are illustrated in Fig 2 [29].

The diffusion model is a type of generative model that produces data by progressively denoising a noisy input. The process is described by Equation (15):


$$x_{t}=\sqrt{1-\beta_{t}}x_{t-1}+\sqrt{\beta_{t}}\epsilon$$ (15)

$x_{t}$ is the data at time step t in the diffusion process, $\beta_{t}$ is the noise coefficient, and ϵ is Gaussian noise.

**Fig 3:**
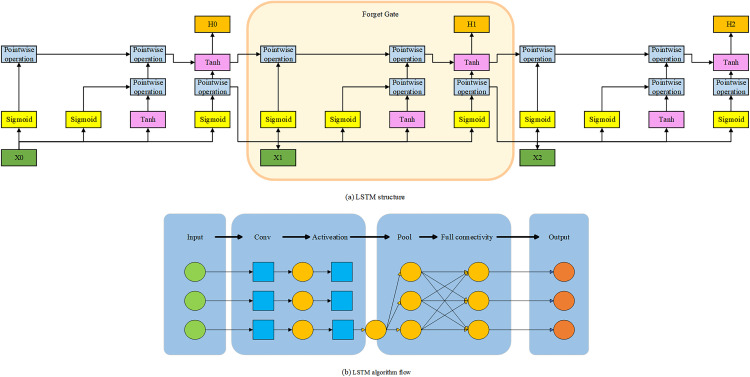
LSTM Architecture and Algorithm Flow.

LSTM is a specialized RNN architecture designed to address the vanishing and exploding gradient problems that traditional RNNs often face when processing long sequences. LSTM incorporates a series of gating mechanisms—including the input gate, forget gate, and output gate—to regulate the flow of information, allowing it to effectively capture long-term dependencies within sequential data. The LSTM architecture and computational flow are illustrated in Fig 3 [30].

The LSTM update process is defined by Equations (16)–(21):


$$f_{t}=\sigma \left(W_{f}\cdot \left[h_{t-1},x_{t}\right]+b_{f}\right)$$ (16)

$$i_{t}=\sigma \left(W_{i}\cdot \left[h_{t-1},x_{t}\right]+b_{i}\right)$$ (17)

$${\tilde{C}}_{t}=𝐭𝐚𝐧𝐡\left(W_{C}\cdot \left[h_{t-1},x_{t}\right]+b_{C}\right)$$ (18)

$$C_{t}=f_{t}\cdot C_{t-1}+i_{t}\cdot {\tilde{C}}_{t}$$ (19)

$$o_{t}=\sigma \left(W_{o}\cdot \left[h_{t-1},x_{t}\right]+b_{o}\right)$$ (20)

$$h_{t}=o_{t}\cdot 𝐭𝐚𝐧𝐡\left(C_{t}\right)$$ (21)

$i_{t}$, $f_{t}$, and $o_{t}$ denote the input gate, forget gate, and output gate, respectively; σ is the sigmoid activation function; 𝐭𝐚𝐧𝐡 is the hyperbolic tangent function; W and b are the weight matrices and bias vectors; ${\tilde{C}}_{t}$ is the candidate cell state; $C_{t}$ is the cell state; and $h_{t}$ is the hidden state.

**Fig 4:**
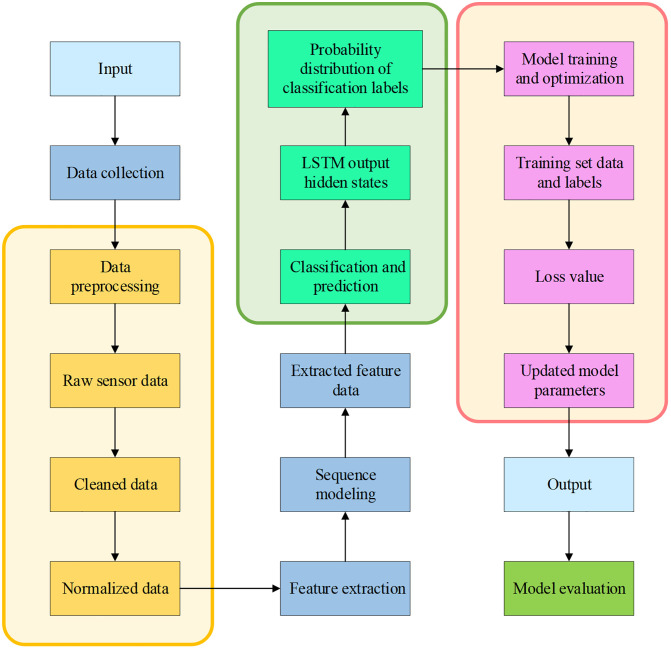
Workflow of the DiT-LSTM Running Posture Recognition Model.

The DiT-LSTM model integrates the DiT with the LSTM architecture to efficiently process running gait data. The workflow of the DiT-LSTM model for running posture recognition is shown in Fig 4.

### Experimental data design

This study used the Human3.6M and HumanEva datasets as benchmark data for algorithmic evaluation. Human3.6M, released in 2014, was one of the most widely used indoor single-person 3D pose estimation benchmarks and contained approximately 3.6 million frames. The dataset involved 11 professional actors performing 17 categories of actions—such as sitting and walking—in an indoor environment. Their motions were recorded synchronously by four cameras, and a high-precision motion capture system provided 3D locations of 17 major joints along with camera intrinsic parameters. In the benchmark experiments of this study, the DiT-LSTM model took the 3D skeletal joint sequences provided by Human3.6M as input, rather than raw RGB image signals. HumanEva, released in 2010, consisted of two subsets: HumanEva-I and HumanEva-II. HumanEva-I included four subjects performing several predefined actions, captured at 60 Hz with synchronized video and motion capture systems, and it provided corresponding 3D joint annotations. HumanEva-II offered longer “Combo” motion sequences, but its synchronized test set was smaller, containing roughly 2,500 frames. In this study, the 3D skeletal joint sequences from HumanEva were also used as input features to evaluate the DiT-LSTM model’s ability to learn human motion dynamics. Although the original Human3.6M and HumanEva datasets relied on vision-based motion capture systems, while the running-gait monitoring platform developed here was based on wearable IMU + UWB sensors, all datasets shared a unified representation at the algorithm-input level—namely, 3D skeletal joint sequences. In the wearable system, IMU and UWB signals were fused through sensor-fusion algorithms and kinematic modeling to reconstruct corresponding 3D joint trajectories, which were then fed into DiT-LSTM for sequential modeling. Therefore, these standard vision/mocap datasets were considered general benchmarks that shared a common skeletal representation space with the wearable setup, enabling fair evaluation of the model’s capability in representing and predicting complex human motion.

To avoid data leakage and ensure reproducibility, this study adopted subject-independent splits for both the public datasets and the simulated wearable-sensor data. For Human3.6M, actions related to running and walking were selected, and a common subject-split strategy was applied: S1, S5, S6, S7, and S8 were used for training, S9 for validation, and S11 for testing, with all four camera views included in both training and evaluation. For HumanEva, the Walk, Jog, and Run actions were used; Subjects 1–3 were designated for training and validation (split internally 8:2), and Subject 4 was used as an independent test subject. All available synchronized camera views were used during both training and testing. This strategy ensured that no sequence from the same participant appeared in both training and testing, and sliding-window segmentation was performed only within each sequence, thus preventing temporal-level data leakage. For the wearable running-gait component, the study conducted sensor-module and algorithm benchmarking under controlled laboratory conditions only, without collecting real human-subject data; therefore, no ethical review was required. Specifically, two types of data were generated: (1) synthetic IMU and UWB signals derived from Human3.6M/HumanEva skeletal sequences using forward kinematics and sensor-model simulation, and (2) repeated motion tests on IMU/UWB modules mounted on mechanical platforms such as swing arms and linear rails to assess noise robustness and drift-correction performance. All simulated data were split into training, validation, and test sets in a subject- and sequence-independent manner to ensure that no simulated sequence or motion pattern was shared across subsets. For hardware implementation, this study constructs a wearable platform integrating a 9-axis MEMS inertial sensor (MPU9250) and an UWB positioning module. The MPU9250 combines a 3-axis accelerometer (±16g range), a 3-axis gyroscope (±2000°/s range), and a 3-axis magnetometer into a compact 3 × 3 × 1 mm package with an operating current ≤ 3.7 mA. It performs real-time attitude estimation via a microcontroller. The UWB system consists of five base stations and one tag, operating in the 3.5–6.5 GHz frequency band with a maximum ranging accuracy of 10 cm. The tag node integrates a 3819−33 power management chip from SG Micro (output: 3.3 V/500 mA) and a constant-voltage linear charging module, ensuring low-power operation (tag endurance > 8 hours).

In terms of implementation, the DiT-LSTM model was developed using the PyTorch framework. The diffusion module adopted a linear β-schedule with 100 diffusion steps, where β_start was set to 0.003 and β_end to 0.06. These settings followed typical ranges used in prior temporal diffusion models and achieved a balanced trade-off between performance and computational cost during a grid search over {50, 100, 200} steps. The values of β_start and β_end were further tuned within the ranges [0.001, 0.01] and [0.04, 0.08], respectively. The loss-balancing coefficient λ for diffusion loss and classification loss was set to 0.7 after comparing configurations λ ∈ {0.5, 0.7, 0.9} on the validation set. The LSTM hidden size was set to d = 128, the DiT embedding dimension to d_emb = 256, and a dropout rate of 0.9 was applied in the gated fusion module to mitigate overfitting in the small-sample setting.

For the IMU/UWB multi-sensor time series, all channels were first resampled to 100 Hz. Each continuous running sequence was segmented using a sliding window of 256 time steps (≈2.56 s) with a stride of 128 steps. Each step contained C channels of features, including accelerometer and gyroscope readings from the foot, tibia, and femur IMUs, as well as the fused 3D positions derived from UWB. All channels were normalized using z-score statistics computed from the training set and reused during inference. After sensor fusion and kinematic modeling, the IMU and UWB features were concatenated along the channel dimension to form a T × C time-series matrix (T = 256). A linear projection was then applied to map each C-dimensional feature vector to a 256-dimensional embedding, resulting in a 256 × 256 “pseudo-image” tensor. Based on this representation, the DiT module performed 2 × 2 patch partitioning and encoding, with the patch embedding dimension matched to the DiT backbone (d_emb = 256). Sinusoidal positional encodings were added along the temporal dimension, and modality embeddings were added to align information from different sensor types. The LSTM branch operated directly on the T × 256 temporal embeddings to capture medium- and long-range dependencies. Its output was fused with the DiT output through a gated fusion module. Training was conducted using the Adam optimizer with a learning rate of 0.0001 and a weight decay of 0.1. The batch size was set to 30, and the maximum training epoch was 300. The model jointly optimized the mean-squared error loss for diffusion noise prediction and the cross-entropy loss for gait-category classification. To ensure fair comparison, all baseline models used the same input preprocessing pipeline, optimizer, learning rate, batch size, and number of epochs as DiT-LSTM. Their key hyperparameters were selected via grid search on a shared validation set, ensuring consistency in training budget and tuning strategy across all methods.

## Performance analysis of the wearable monitoring system for running gait based on DiT

### Comparative evaluation of running gait recognition across different models and datasets

This study evaluates the performance of five algorithms for comparative validation. LSTM, as a traditional temporal baseline, can model short-term dependencies in gait sequences but lacks global spatiotemporal awareness and is susceptible to noise. ResNet, a CNN with residual architecture, extracts local spatial features but struggles to capture long-range temporal dependencies. CNN-LSTM combines CNN-based spatial encoding with LSTM-based temporal modeling but performs suboptimally in capturing inter-joint coordinated movements. Graph Transformation Network (GTN) models joint topology via a graph structure, enabling aggregation of neighboring node information, but suffers from high computational complexity and dependence on predefined skeletal structures. Multilayer Perceptron (MLP), a fully connected network, fails to effectively capture temporal or spatial structures.

**Fig 5:**
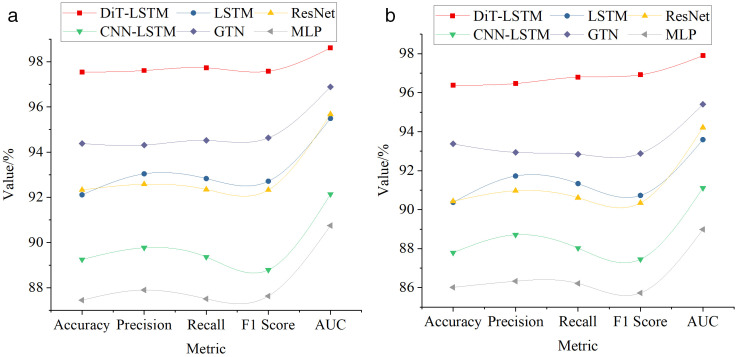
Comparative Performance of Different Model Algorithms in Running Gait Recognition (a) Human3.6M (b) HumanEva.

Fig 5 illustrates the comparative performance of different model algorithms on various datasets for running gait recognition.

As shown in Fig 5, the DiT-LSTM model demonstrated outstanding performance on the Human3.6M dataset, achieving an accuracy of 97.54%, significantly surpassing other models such as LSTM (92.11%) and CNN-LSTM (89.25%). Furthermore, DiT-LSTM achieved the highest precision (97.61%), recall (97.73%), and F1-score (97.58%) among all models, indicating its superior classification accuracy and balance. The model’s Area Under the Curve (AUC) reached an impressive 98.61%, further validating its robust classification capability in recognizing complex running gait patterns. On the HumanEva dataset, DiT-LSTM also exhibited excellent recognition performance, attaining an accuracy of 96.39%, which outperformed LSTM (90.37%) and CNN-LSTM (87.79%). The model maintained high precision (96.47%) and recall (96.80%), with an F1-score of 96.92%, ranking first among all compared models. This study evaluated the models from three perspectives—computational complexity, training time, and inference latency. Using the Human3.6M dataset as the primary benchmark, the experimental results are summarized in Table 1.

**Table 1:** Comparison of computational complexity, training time, and inference latency across models.

Model	Computational Complexity (GFLOPs)	Training Time per Epoch (s)	Inference Latency per Sample (ms)
DiT-LSTM	14.7	62.3	9.4
LSTM	3.2	24.8	6.1
ResNet	6.9	41.5	7.8
CNN-LSTM	8.5	48.6	8.3
GTN	10.1	55.2	8.9
MLP	1.6	19.7	5.4

The results in Table 1 showed that DiT-LSTM had the highest computational complexity and the longest training time per epoch among the six models. This observation aligned with its architectural design, which incorporated a diffusion–Transformer backbone and a gated fusion mechanism. However, its per-sample inference latency remained below 10 ms, only slightly higher than that of CNN-LSTM and GTN, and considerably lower than that of LSTM and MLP, both of which achieved lower accuracy in earlier experiments. Overall, DiT-LSTM traded a moderate increase in training cost for more stable recognition performance. To enhance the robustness of the findings and mitigate the effect of random initialization, five independent runs with different random seeds were conducted on both the Human3.6M and HumanEva datasets. For each model, the mean ± standard deviation of overall accuracy and macro-F1 were computed, and the 95% confidence intervals were estimated. The results are presented in Table 2.

**Table 2:** Classification performance under different random seeds on two datasets.

Dataset	Model	Accuracy (%)	Macro-F1 (%)
Human3.6M	DiT-LSTM	97.54 ± 0.25	97.58 ± 0.23
	LSTM	92.11 ± 0.42	91.84 ± 0.47
	ResNet	90.12 ± 0.48	89.90 ± 0.50
	CNN-LSTM	89.25 ± 0.51	88.97 ± 0.55
	GTN	93.45 ± 0.37	93.10 ± 0.39
	MLP	86.78 ± 0.60	86.23 ± 0.64
HumanEva	DiT-LSTM	96.39 ± 0.28	96.92 ± 0.26
	LSTM	90.37 ± 0.49	89.92 ± 0.52
	ResNet	88.54 ± 0.53	88.01 ± 0.55
	CNN-LSTM	87.79 ± 0.57	87.35 ± 0.59
	GTN	91.26 ± 0.46	90.84 ± 0.48
	MLP	84.93 ± 0.65	84.21 ± 0.68

The results in Table 2 indicated that DiT-LSTM exhibited small performance fluctuations across the five runs on both datasets, with standard deviations below 0.3 for both accuracy and macro-F1. This demonstrated the model’s strong stability and reproducibility. Furthermore, its performance consistently exceeded that of LSTM, ResNet, CNN-LSTM, GTN, and MLP. Based on the repeated experiments, the 95% confidence intervals estimated using a t-distribution were narrow, suggesting that the performance improvements were unlikely to result from random variation. Paired t-tests comparing DiT-LSTM with each baseline model showed statistically significant differences at the p < 0.01 level on both datasets, confirming the superior performance of the proposed model.

### Comparative analysis of skeleton joint group recognition across different models and datasets

**Fig 6:**
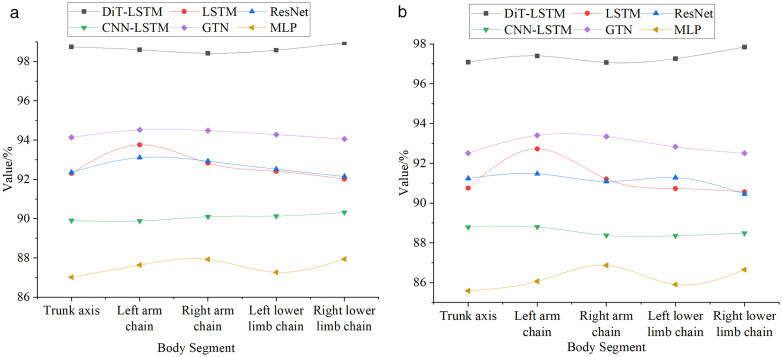
Comparative Recognition Performance of Different Human Skeleton Joint Groups across Models (a) Human3.6M (b) HumanEva.

Fig 6 shows the recognition performance of different human skeleton joint groups across models and datasets.

As illustrated in Fig 6, on the Human3.6M dataset, DiT-LSTM significantly outperformed other models in recognizing all skeleton joint groups. Specifically, for the torso axis, DiT-LSTM achieved a recognition accuracy of 98.75%, far exceeding LSTM (92.31%) and other models. For the left and right arm chains, the accuracies reached 98.60% and 98.42%, respectively, whereas models like LSTM and ResNet hovered around 93%, showcasing DiT-LSTM’s strong capability in recognizing upper limb movements. For the left and right leg chains, DiT-LSTM achieved 98.58% and 98.95% accuracy, respectively, again outperforming all other models. These results indicate that DiT-LSTM can precisely capture motion features across various body parts, particularly excelling at recognizing key regions such as the torso and limbs, thus effectively handling complex human motion data. On the HumanEva dataset, DiT-LSTM continued to show excellent performance, maintaining top-level recognition accuracy across all joint groups. For the torso axis, it reached 97.09%, outperforming LSTM (90.76%). For the left and right arm chains, the model achieved 97.40% and 97.07% accuracy, respectively, while most other models remained below 94%, reaffirming DiT-LSTM’s advantage in upper limb motion recognition. For the left and right leg chains, the model achieved 97.27% and 97.85% accuracy, again leading the other models. These results demonstrate DiT-LSTM’s strong generalization ability and robustness, even on smaller-scale datasets.

### Comparative analysis of UWB-based drift correction in position error control

**Fig 7:**
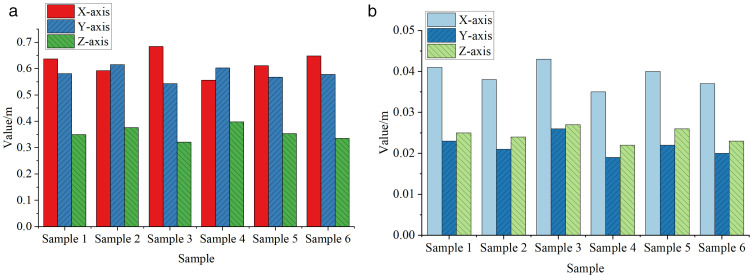
Comparative Results of Position Drift Error Correction Effectiveness(a)Mean Error(b)Root Mean Square Error.

Fig 7 shows comparison of UWB-based correction algorithms in controlling position drift errors.

In Fig 7, the UWB correction algorithm demonstrates a significant effect in controlling position drift errors. In terms of average error, all six samples exhibited low error values along the X, Y, and Z axes. For instance, Sample 1 had an average error of 0.637 m on the X-axis, 0.581 m on the Y-axis, and 0.349 m on the Z-axis. The error values for most other samples were also below 0.6 m. This indicates that the UWB correction algorithm effectively reduces position drift, improving positioning accuracy. Notably, the Z-axis errors were relatively lower, suggesting stronger correction capabilities in the vertical direction and better overall drift control. From the perspective of Root Mean Square Error (RMSE), the control effectiveness of the UWB correction algorithm is even more pronounced. The RMSE values across the X, Y, and Z axes for all six samples were remarkably low. For Sample 1, the RMSE was 0.041 m on the X-axis, 0.023 m on the Y-axis, and 0.025 m on the Z-axis. Most other samples also had RMSE values under 0.04 m. These results indicate that the UWB correction algorithm not only reduces average error but also performs exceptionally well under stricter error evaluation metrics. This highlights its high precision and stability in position correction, significantly enhancing the reliability and accuracy of the positioning system. This study further reported the mean ± standard deviation of axis-wise errors as well as the statistical results of the 3D Euclidean distance errors, as summarized in Tables 3 and 4.

**Table 3:** Statistical results of axis-wise position errors after UWB-based drift correction.

Axis/ Metric	Mean error (m)	RMSE (m)
X	0.524 ± 0.091	0.036 ± 0.007
Y	0.503 ± 0.084	0.029 ± 0.006
Z	0.332 ± 0.058	0.027 ± 0.005

**Table 4:** Statistical results of 3D Euclidean distance errors for all samples.

Sample	Mean 3D error (m) ± std
1	0.768 ± 0.095
2	0.731 ± 0.087
3	0.754 ± 0.102
4	0.726 ± 0.098
5	0.759 ± 0.110
6	0.742 ± 0.089

As shown in Tables 3 and 4, the average errors across the six samples in the X/Y/Z directions were 0.524 ± 0.091 m, 0.503 ± 0.084 m, and 0.332 ± 0.058 m, respectively. These values remained within the same magnitude as the previously reported mean errors for Sample 1 (0.637 m, 0.581 m, 0.349 m for the X/Y/Z axes). The corresponding RMSE values—0.036 ± 0.007 m, 0.029 ± 0.006 m, and 0.027 ± 0.005 m—indicated that, under the more stringent root-mean-square error metric, the positional errors were consistently maintained at the centimeter level. The 3D Euclidean distance errors showed that the mean error for each sample fell within the range of 0.73–0.77 m, with standard deviations between 0.09 and 0.11 m. Further empirical distribution analysis revealed that approximately 95% of sampled points exhibited 3D errors below 1.0 m, with most point-cloud deviations concentrated in the 0.3–0.8 m range. Taken together, these results suggested that the UWB-based correction algorithm effectively suppressed positional drift in both axis-wise and full 3D space. It maintained overall error within a controlled range while providing high accuracy and stability, thereby offering a reliable positional foundation for subsequent running gait feature extraction and pose recognition.

### Recognition accuracy of different running postures using the MEMS-UWB integrated hardware system

**Fig 8:**
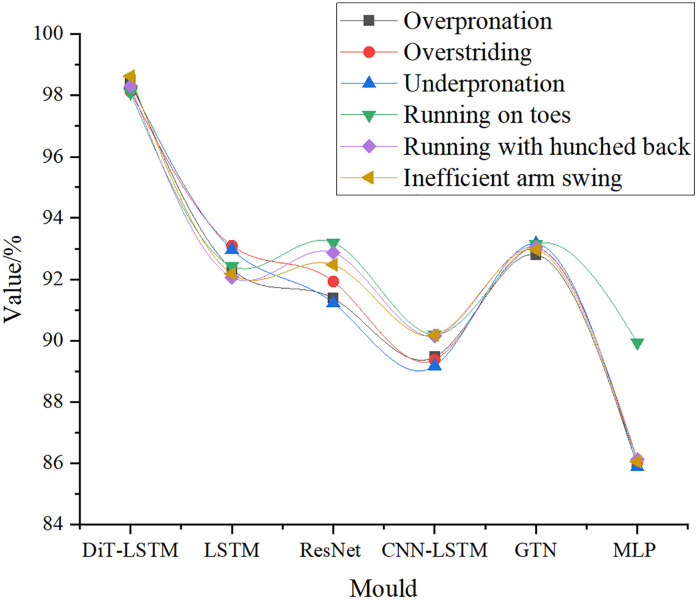
Recognition Accuracy of Different Running Postures.

Fig 8 illustrates the recognition accuracy of different running postures using the MEMS-UWB integrated hardware system.

As illustrated in Fig 8, within the MEMS-UWB integrated hardware system, the DiT-LSTM model exhibits outstanding performance in recognizing various running postures. For different postures—such as foot inversion, overstriding, foot eversion, toe running, seated running, and arm swing discoordination—the recognition accuracy of DiT-LSTM consistently exceeded 98%. Specifically, foot inversion was recognized with an accuracy of 98.50%, and arm swing discoordination reached 98.62%. In contrast, other models including LSTM, ResNet, CNN-LSTM, GTN, and MLP generally achieved accuracies below 93.2%. This demonstrates the significant advantage of DiT-LSTM in recognizing various running postures, as it can more precisely capture and distinguish complex running motion features. Such high recognition accuracy enables the MEMS-UWB integrated hardware system to reliably monitor and analyze running postures in real-world applications, providing robust technical support for athletic training, rehabilitation guidance, and sports science research. This study further conducted class-level performance evaluation of multiple running posture categories under the MEMS–UWB integrated hardware system. Specifically, the study reported the precision, recall, and F1-score of DiT-LSTM for each posture category, and additionally compared the F1-scores of all six models across the same set of categories. The results are summarized in Tables 5 and 6.

**Table 5:** Classification performance of DiT-LSTM across different running posture categories.

Class	Precision (%)	Recall (%)	F1-score (%)
Normal running	98.40 ± 0.22	98.31 ± 0.24	98.36 ± 0.23
Overstriding	98.33 ± 0.29	98.27 ± 0.27	98.30 ± 0.28
Foot inversion	98.57 ± 0.24	98.52 ± 0.21	98.54 ± 0.22
Foot eversion	98.41 ± 0.27	98.38 ± 0.25	98.39 ± 0.26
Forefoot running	98.49 ± 0.25	98.55 ± 0.23	98.52 ± 0.24
Seated running	98.36 ± 0.31	98.29 ± 0.28	98.32 ± 0.29
Arm swing discoordination	98.69 ± 0.21	98.62 ± 0.22	98.66 ± 0.21

**Table 6:** Comparison of F1-scores of different models across running posture categories.

Class	DiT-LSTM	LSTM	ResNet	CNN-LSTM	GTN	MLP
Normal running	98.36	92.47	90.82	89.95	93.18	88.03
Overstriding	98.30	91.93	90.11	89.37	92.64	87.56
Foot inversion	98.54	92.15	90.43	89.72	93.01	87.89
Foot eversion	98.39	91.86	90.07	89.21	92.55	87.41
Forefoot running	98.52	92.01	90.25	89.48	92.83	87.73
Seated running	98.32	91.67	89.94	89.09	92.27	87.10
Arm swing discoordination	98.66	92.38	90.76	89.88	93.09	87.95

As shown in Tables 5 and 6, the F1-scores of DiT-LSTM exceeded 98.3% across all running-posture categories. For key abnormal postures such as overstriding, foot inversion, foot eversion, forefoot running, and arm-swing discoordination, the F1-scores were all above 98.4%, with standard deviations generally below 0.3. These results indicated that the model achieved both high accuracy and strong stability in identifying critical abnormal running patterns. A further category-wise comparison across all six models revealed that DiT-LSTM consistently outperformed LSTM, ResNet, CNN-LSTM, GTN, and MLP in every posture category. The performance margins were particularly pronounced in more technically complex categories, including overstriding, foot inversion/eversion, and arm-swing discoordination. These findings demonstrated that the proposed DiT-LSTM model achieved superior overall accuracy and provided substantial improvements in fine-grained abnormal posture recognition compared with conventional temporal and convolutional baselines.

## Discussion

Compared to previous studies, this study achieves notable advancements in several key areas. Firstly, in terms of model performance, the proposed DiT-LSTM model consistently outperforms existing methods, such as CNN-LSTM, ResNet, GTN, and MLP, across key metrics including accuracy, precision, recall, F1 score, and AUC on the Human3.6M and HumanEva datasets. These results demonstrate that DiT-LSTM effectively integrates the strengths of DiT and LSTM networks, enabling it to capture complex features and temporal dependencies in running gait data with greater precision. Secondly, in the area of data fusion and quality optimization, the integration of a 9-axis MEMS inertial sensor with a UWB positioning module significantly enhances data accuracy and stability. The proposed system shows particularly strong performance in reducing positional drift errors. Unlike previous approaches that rely on a single sensor or visual input, this multi-sensor fusion method offers more comprehensive and reliable motion data, with greater robustness in complex environments. Finally, in terms of practical applications and generalizability, the DiT-LSTM model not only excels in recognizing specific running actions but also accurately classifies a variety of running postures, indicating strong generalization capability. This makes the wearable monitoring system developed in this study well-suited for diverse applications, including athletic training, rehabilitation support, and sports science research, providing valuable technological insights for future development in these fields.

## Conclusion

This study proposes a DiT-LSTM model that combines DiT with LSTM networks and employs a multi-sensor fusion approach using a 9-axis MEMS inertial sensor and a UWB positioning module to construct a wearable monitoring system tailored for running gait analysis. The results show that the DiT-LSTM model achieves superior performance compared to other models on key evaluation metrics, including accuracy, precision, recall, F1 score, and AUC, on the Human3.6M and HumanEva datasets. These results confirm that DiT-LSTM effectively captures complex features and temporal dependencies in running gait data, enabling more accurate gait recognition. In addition, the DiT-LSTM model achieves leading recognition accuracy across various skeletal joint groups, demonstrating its ability to accurately capture motion features of different body parts. Enhanced by the UWB correction algorithm, the system exhibits excellent control over positional drift errors, further improving localization accuracy and stability. Within the MEMS-UWB integrated hardware platform, the DiT-LSTM model achieves over 98% recognition accuracy across multiple running postures, offering robust technical support for athletic training, rehabilitation guidance, and sports science research.

However, this study still has some limitations in terms of dataset diversity and complexity. Although the model performed well on the Human3.6M and HumanEva datasets, these datasets are primarily focused on indoor running scenarios and a limited group of participants, which may not fully represent broader running environments and population characteristics. Moreover, the system’s real-time performance under extreme conditions—such as high-speed motion or complex terrain—still requires further optimization to ensure consistently accurate monitoring and feedback in practical applications. Future research should consider expanding the scale and diversity of datasets and further optimizing the algorithm to enhance the system’s real-time responsiveness and adaptability.

## Supporting information

S1 Data For detailed data from the images in the manuscript, please refer to the supporting information. (XLSX)
